# An In Vitro Comparison of Propex II Apex Locator to Standard Radiographic Method

**Published:** 2013-08-01

**Authors:** Kalyan Vinayak Chakravarthy Pishipati

**Affiliations:** a Department of Conservative Dentistry and Endodontics, Penang International Dental College, Malaysia

**Keywords:** Apex Locator, Endodontics, Periapical Radiographly, Working Length

## Abstract

**Introduction:**

The aim of this in vitro study was to compare the accuracy of radiography in assessing working length to Propex II apex locator.

**Materials and Methods:**

Thirty single canal extracted human teeth with patent apical foramen were selected. Access cavities were prepared. Anatomic length (AL) was determined by inserting a K-file into the root canal until the file tip was just visible at the most coronal aspect of the apical foramen; subsequently 0.5 mm was deducted from this measured length. Working length by radiographic method (RL) was determined using Ingle’s method. Propex II apex locator was used to determine the electronic working length (EL). From these calculated lengths, AL was deducted to obtain D-value. D-value in the range of +/-0.5 mm was considered to be acceptable.

**Results:**

The percentage accuracy of RL and Propex II apex locator was 76.6% and 86.6%, respectively. Paired t-test revealed significant difference between the RL and Propex II apex locator (P<0.05).

**Conclusion:**

Under these in vitro conditions, Propex II apex locator has determined working length more accurately than radiographic method.

## Introduction

Determination and maintainance of accurate working length are critical steps in endodontic therapy. Failure to accurately determine and maintain the working length might result in over/under filling and subsequent failure of root canal treatment [1]. Traditionally, working length was determined by radiographic method but it had obvious drawbacks. The position of the apical constriction or the major foramen can not be detected [2-5] and it provided a two dimensional image of a three dimensional object [2, 3]. Also, superimposition of bony structures hindered the identification of radiographic apex of some teeth [6]. Radiographic method is technique sensitive and is subjected to operator interpretation and quality issues like distortion and magnification. In addition, some patients may express radiation concerns.

To overcome these shortcomings, electronic method of working length determination was developed and is rapidly gaining popularity and use. It was conceptualized by Custer [7] and later revisited by Suzuki [8] in 1942 who observed that a consistent electrical resistance between an instrument in a root canal and an electrode on oral mucous membrane could be used for measuring canal length. Since that discovery, several generations of electronic apex locators (EAL) have been developed. First generation of EAL was resistance based whereas the second generations were based on impedance. Both these types had low accuracy in the presence of fluids in the canal [9]. Third generation EAL’s were frequency based used multiple frequencies to determine the position of file tip in the canal [10]. Later, fourth generation devices were developed which measure resistance and capacitance separately (rather than the resultant impedance value) for greater accuracy [11]. Propex II (Dentsply Maillefer, Tulsa, OK, USA) is multi-frequency based fifth generation apex locator that uses multiple frequencies to determine the root canal length. Rather than using the amplitude of the signal as for all EALs, it measures the energy of the signal with multi signal frequencies. As there were few studies in this field, the objective of this *in vitro* study was to compare the accuracy of radiographic method and Propex II apex locator.

## Material and Methods

Thirty extracted single canal teeth with patent apical foramen were used in this study. The selected teeth were free of any obvious caries, previous restorations, open apices, resorptive defects or root canal treatments. Teeth were kept in 1 % thymol solution for one day. They were cleaned with hand scalar to remove calculus. Teeth were numbered 1-30 for easy identification. Access cavities were prepared and cervical portions of root canal were flared using #2 and #3 Gates-Glidden drills (Dentsply, Maillefer, Tulsa, USA). Thorough irrigation was performed with 5% sodium hypochlorite (Prime Dental Products Private Limited, Mumbai, India) by using blunt needle which was placed as deep as possible without obstructing the root canal.

A #6 K-file (Dentsply, Maillefer, Tulsa, OK, USA) was inserted into the root canal until the file was just visible at the most coronal aspect of the apical foramen when viewed under 50× magnification by Unitron Z850 series stereo microscope (Unitron, Commack, NY, USA) and the silicone stopper was adjusted to coronal/ incisal reference plane to this length. The distance between file tips to the silicone stopper was measured with digital vernier calliper (Mitutoyo, Tokyo, Japan). From this length 0.5 mm was deducted to obtain Anatomic length (AL).

Each tooth was then mounted in a plastic template box filled with addition silicone elastomeric material (3M ESPE, USA) allowing easy removal and fixation of tooth in reproducible position. The template was a plastic box with a base dimension corresponding to the size #2 of an intra-oral periapical film Kodak E-speed film (Eastman Kodak Co., Rochester, NY, USA). The film was placed in contact with base of the template within its confines. All radiographs were taken using X-ray generator (Unicorn DenMart, New Delhi, India) whichwas set at 70 Kv, 8 mA and exposed for 0.4 sec with source object distance of 20 cm. X-ray films were developed and viewed using a standard viewing box under 4× magnification .

Radiographic working length determination was performed with Ingle’s Method. A #15 K-file with 1 mm less length than the tooth length (safety factor), as noted from the preoperative radiograph, was kept in the root canal and radiograph was taken. On the radiograph, the difference between the end of the file and the apex was measured. This amount was added or subtracted to the original measured length. From this adjusted length of tooth, 1 mm was subtracted to confirm with the cementodentinal junction. This value was registered as radiographic length (RL).

Each tooth was mounted in a metal ring that held the tooth. A device with digital micrometer read out Instron universal testing machine (Instron Corporation, Canton, MA, USA) which moved attached #15 K-file with a precision of 0.1 mm was used. The file clip of Propex II apex locator was attached to the file and the lip clip was immersed in a container holding electro-conductive medium (normal saline). The apex of the tooth with inserted file was in contact with normal saline completing the circuit. The file was lowered gradually till the device display showed 0.0 or apex. The silicon stopper was adjusted, and the length was measured using a digital vernier calliper (Mitutoyo, Japan). This was termed as electronic length (EL).

Anatomic length (AL) was determined to provide a base line data against which measurements by radiographic method (RL) and Propex II apex locator (EL) could be compared. In present study, D-values were calculated, i.e. the difference between working length determined by radiographic (RL), Propex II apex locator (EL) and anatomic length (AL). D-value in range of +/-0.5 mm was considered acceptable and percentage accuracy by radiographic method and Propex II apex locator was calculated. D-values obtained by radiographic method and Propex II apex locator were compared using a paired sample t-test.

## Results

The acceptable measurements of radiographic method and Propex II apex locator were 76.6% and 86.6%, respectively. Over estimation of working length determination by radiographic method and Propex II apex locator were 20% and 3.33%, respectively. Underestimations of working length determination by radiographic method and Propex II apex locator were 3.33% and 10%, respectively (Table 1). The mean D-value with radiographic method was 0.263 mm and with Propex II apex locator was 0.12 mm (Table 2). There was significant difference between radiographic method and Propex II apex locator (*P*<0.024) (Table 3).

**Table 1.** Percentage accuracy

	Acceptable Measurement D-value within =/- 0.5mm	Overestimation D-value> 0.5 mm	Underestimation D-value<- 0.5 mm
**Radiographic method**	76.66%(23)	20.00%(6)	3.33%(1)
**Propex II**	86.66%(26)	3.33%(1)	10.00%(3)

**Table 2.** Paired samples statistics

	Mean (SD)	N	SE
**Radiographic method**	0.42 (0.26)	30	0.07
**Propex II**	0.38(0.12)	30	0.06

**Table 3.** Paired Samples Test

	Mean (SD)	SE	95% CI		t	Df	Sig.
			Lower	Upper			
**Radiographic method-Propex II**	0.14 (0.33)	0.06	0.025	0.261	2.37	29	0.02

## Discussion

Accurate determination of working length is a key factor in successful endodontic treatment. Several modalities can be employed for this purpose which has its own merits and demerits. In our study, the accuracy of Propex II apex locator and radiographic method was compared with the AL base line data. For this a #6 K-file was inserted with file tip just visible at the coronal aspect of apical foramen under 50× magnification. A #6 K-file was used for anatomic measurement of the apex to avoid alteration of apical anatomy under 50× magnification [3]. All visual measurements were made from the most coronal aspect of major diameter because it was reproducible and consistent [3, 12-14]. AL measurements were derived by deducting 0.5 mm from measured file length in accordance to studies of Kutler [15] which states that apical constriction on an average is 0.5-0.7mm short of the major diameter. Kutler’s view has been confirmed by other studies [16]. Radiographic method is the most common technique of working length determination. We used Ingle’s method [17], which is considered most acceptable radiographic method. Radiographs were taken using individual template for each tooth in combination with paralleling technique. This assists in the reproducibility of the radiograph technique and reduces the potential interpretation errors [3].

*In vitro* studies like ours, use electro-conductive materials to simulate the clinical situation [18]. Various materials like alginate [19], agar [20], saline [4, 14] and gelatin [21] have been used as electro-conducting medium in different studies. It has been suggested that electronic apex locators operate on the principle of electricity rather than biological properties of tissues involved. Therefore the models in which the extracted teeth are immersed in media with electric resistance similar to that of periodontal ligament tissue can give precise and reliable information on their function [22]. However, some of these media can leak through the apical foramen and cause premature readings [23].

In the present study D-values were calculated. D Value in the range of +/-0.5mm was considered clinically acceptable range [9]. The present study indicates that the acceptable measurements of radiographic and Propex II apex locator were 76.66% and 86.6%, respectively. Several studies have indicated a higher level of accuracy of apex locators compared to radiographic method [3, 24, 25]. In this study Propex II apex working length estimation was 86.6% accurate considering 0.5 mm tolerance, which concurs with previous *in vitro* studies: Cianconi *et al*., 83.2% [26]; Karunakar *et al., *85% [27]; and Paul *et al*., 82.1% [28]. In Kqiku *et al.* *in vitro* study [29] of Propex II was found to be 93.4% accurate in determining working length. In an *in vivo* study, with low number of samples (*n*=10), Propex II produced 4 acceptable, 5 long and 1 short measurement indicating an accuracy of only 40% [30]. However as this was an *in vivo *study with a low number of samples it is difficult to compare it with our study and draw conclusions. According to Srinivasan *et al. *[31] and Yadav *et al*. [32], Propex II was able to detect simulated oblique root fracture with an accuracy of 63.3% and 53.4% respectively when 0.5 mm tolerance was used.

Propex II apex locator was more accurate in detecting the apical foramen in bicuspids than in molars and anterior teeth [33]. Morgental *et al. *reported increased accuracy of Propex II apex locator after pre-flaring the canal [34]. Moreover, the accuracy of Propex II apex locator was affected by the size of the file used and was able to locate the physiologic foramen (apical constriction) with an accuracy of 38.62%, 45% and 40.63% when #08, 10 and 15 K-files were used [35].

In our study, overestimation of working length determination by radiographic and Propex II apex locator are 20% and 3.33%, respectively. Further studies have shown that Propex II has 75% accuracy when determining minor constriction, 20% short and 5% beyond minor constriction whereas radiographic method was 10% accurate, 45% short and 45% beyond minor constriction [36]. Electronic working lengths were superior to radiographs in reducing the overestimation of root canal length [6, 25, 37]. Using an electronic apex locator as an aid to endodontic therapy could also potentially reduce the number of diagnostic radiographs required for working length determination [38, 39].

## Conclusion

Under the *in vitro* conditions, Propex II was more accurate than the radiographic method in determining working length. Apex locator can reduced the overestimation observed in radiographic method.
